# Design and Synthesis of Arylpiperazine Serotonergic/Dopaminergic Ligands with Neuroprotective Properties

**DOI:** 10.3390/molecules27041297

**Published:** 2022-02-15

**Authors:** Margherita Mastromarino, Mauro Niso, Carmen Abate, Ewgenij Proschak, Mariam Dubiel, Holger Stark, Marián Castro, Enza Lacivita, Marcello Leopoldo

**Affiliations:** 1 Dipartimento di Farmacia-Scienze del Farmaco, Università degli Studi di Bari Aldo Moro, Via Orabona 4, 70125 Bari, Italy; margherita.mastromarino@uniba.it (M.M.); mauro.niso@uniba.it (M.N.); carmen.abate@uniba.it (C.A.); marcello.leopoldo@uniba.it (M.L.); 2 Institute of Pharmaceutical Chemistry, Goethe-University, Max-von-Laue-Str. 9, 60438 Frankfurt am Main, Germany; proschak@pharmchem.uni-frankfurt.de; 3 Institute of Pharmaceutical and Medicinal Chemistry, Heinrich Heine University Düsseldorf, Universitaetsstrasse 1, 40225 Duesseldorf, Germany; mariam.dubiel@hhu.de (M.D.); stark@hhu.de (H.S.); 4 Center for Research in Molecular Medicine and Chronic Diseases (CIMUS), Universidade de Santiago de Compostela, Avda de Barcelona s/n, 15782 Santiago de Compostela, Spain; marian.castro@usc.es

**Keywords:** serotonin, dopamine, 5-HT_1A_, 5-HT_2A_, 5-HT_7_, D_2_, oxidative stress, arylpiperazine

## Abstract

Long-chain arylpiperazine scaffold is a versatile template to design central nervous system (CNS) drugs that target serotonin and dopamine receptors. Here we describe the synthesis and biological evaluation of ten new arylpiperazine derivatives designed to obtain an affinity profile at serotonin 5-HT_1A_, 5-HT_2A_, 5-HT_7_ receptor, and dopamine D_2_ receptor of prospective drugs to treat the core symptoms of autism spectrum disorder (ASD) or psychosis. Besides the structural features required for affinity at the target receptors, the new compounds incorporated structural fragments with antioxidant properties to counteract oxidative stress connected with ASD and psychosis. All the new compounds showed CNS MultiParameter Optimization score predictive of desirable ADMET properties and cross the blood–brain barrier. We identified compound **12a** that combines an affinity profile compatible with antipsychotic activity (5-HT_1A_ *K*_i_ = 41.5 nM, 5-HT_2A_ *K*_i_ = 315 nM, 5-HT_7_ *K*_i_ = 42.5 nM, D_2_ *K*_i_ = 300 nM), and compound **9b** that has an affinity profile consistent with studies in the context of ASD (5-HT_1A_ *K*_i_ = 23.9 nM, 5-HT_2A_ *K*_i_ = 39.4 nM, 5-HT_7_ *K*_i_ = 45.0 nM). Both compounds also had antioxidant properties. All compounds showed low in vitro metabolic stability, the only exception being compound **9b**, which might be suitable for studies in vivo.

## 1. Introduction

**Figure 1:**
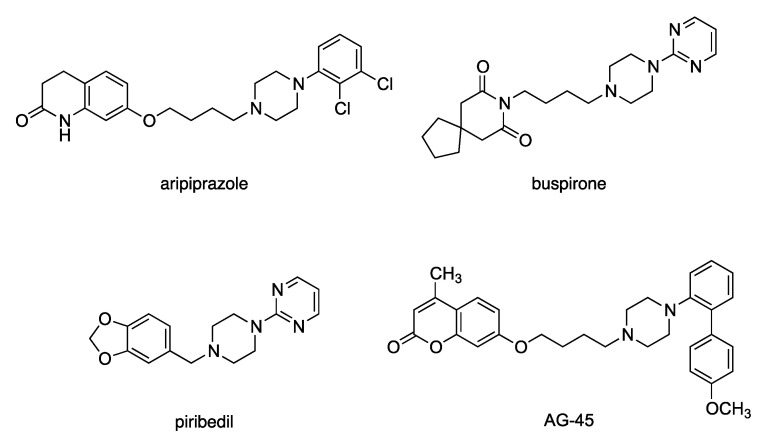
Representative arylpiperazine derivatives.

The long-chain arylpiperazine structure is a versatile model that has allowed the identification of several drugs over the years, such as antipsychotics (aripiprazole, ziprasidone, and lurasidone) [1], anxiolytics (buspirone, tandospirone) [2], and antiparkinsonian drugs (piribedil) [3] (Figure 1). This variety of actions is due to the possibility of modulating the pharmacological profile of the arylpiperazine derivatives through structural variations. Even if more than 30 years have passed since the introduction of buspirone in the market, the long-chain arylpiperazine model is still valid, so that, in the last five years, new drugs with this structure have been introduced on the market [4].

The above-mentioned drugs owe their action to the modulation at the central nervous system (CNS) level of one or more serotonin and/or dopamine receptors that are relevant to the specific pathology. For example, aripiprazole owes its antipsychotic action to the blocking of dopaminergic D_2_ and serotonergic 5-HT_2A_ receptors. The anxiolytic action of buspirone is due to a partial agonist activity on the 5-HT_1A_ receptors, while the antiparkinsonian effect of piribedil is due to dopaminergic D_2_ receptors activation. Recently, we reported on a set of arylpiperazine derivatives with an activity profile on a subgroup of serotonin receptors (5-HT_7_ and 5-HT_1A_ agonist and 5-HT_2A_ antagonist) specifically designed to obtain prospective drugs for the treatment of the core symptoms of autism spectrum disorder [5]. Over the last few years, several studies have highlighted that besides an adequate modulation of the neuroreceptors relevant in the pathology, a neuroprotective action due to a reduction of oxidative stress might have a beneficial effect on these pathologies. In fact, schizophrenia, depression, and autism spectrum disorder (ASD) have a significant neuroinflammatory component linked to oxidative stress [6]. Therefore, the advantage of intervening in targeting a set of receptors and oxidative stress is evident. Of note, recent studies have shown that the activation of the 5-HT_1A_ [7] and 5-HT_7_ receptors [8] produces neuroprotective effects. Thus, for the present study, we have designed a set of new arylpiperazine derivatives with the structural characteristics to target a group of neuroreceptors relevant to treating schizophrenia or ASD and producing neuroprotective effects.

## 2. Results

### 2.1. Study Design

**Figure 2:**
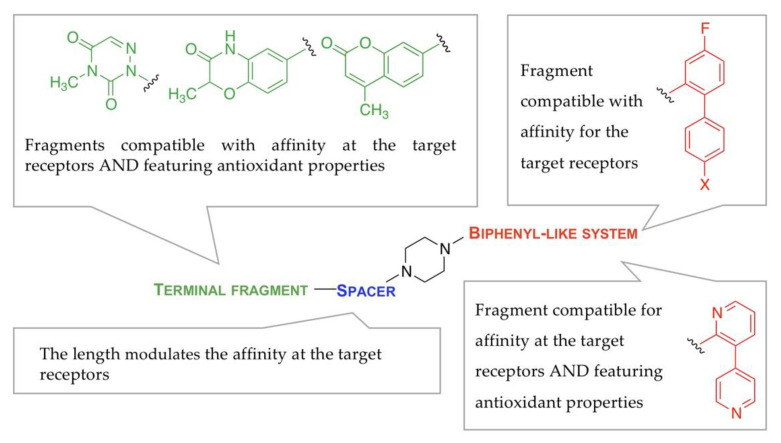
Design Strategy for the Target Compounds.

The starting point for our study was AG-45 (compound 20b in Ref. [5], Figure 1), which has an affinity for 5-HT_1A_, 5-HT_2A_, and 5-HT_7_ receptors. We specifically searched this affinity profile to obtain prospective drugs for the treatment of the core symptoms of ASD. Thus, we introduced structural fragments known for their antioxidant properties, such as the pyridyl [9], 1,2,4-triazine [10], 1,4-benzoxazine [11], and benzopyrone [12], and left unchanged the features responsible for the affinity profile (i.e., the biphenyl-like structure linked to the piperazine ring, and a trimethylene or tetramethylene spacer between the piperazine ring and the terminal fragment). The design of the target compounds is graphically illustrated in Figure 2. Interestingly, AG-45 showed a measurable affinity for the D_2_ receptor, and this prompted us to evaluate structural modifications to increase D_2_ receptor affinity in the prospect of obtaining antipsychotic compounds with antioxidant properties. Of note, while the affinity for D_2_ and 5-HT_2A_ receptors is a crucial feature of atypical antipsychotics, targeting 5-HT_1A_ and 5-HT_7_ receptors have been variously correlated to beneficial effects in the context of psychosis [13,14].

The discovery of CNS drugs also faces the challenge of controlling physicochemical properties. CNS Multi-Parameter Optimisation (MPO) scoring tool is an approach that can facilitate the design of CNS drugs [15]. This tool gives a desirability score (0.05–1) for six physicochemical properties: molecular weight (MW); calculated partition coefficient (cLog*P*); calculated distribution coefficient at pH = 7.4 (cLog*D*_7.4_); acid dissociation constant (pK_a_); topological polar surface area (TPSA); number of hydrogen bond donors (HBD). The sum of these scores yields the CNS MPO score on a 0.3–6 scale. A CNS MPO desirability score higher than 4 is predictive of desirable ADMET properties and cross the blood–brain barrier [15]. As shown in Table 1, all the newly designed compounds display favorable characteristics for a prospective CNS drug.

### 2.2. Chemistry

**Scheme 1:**
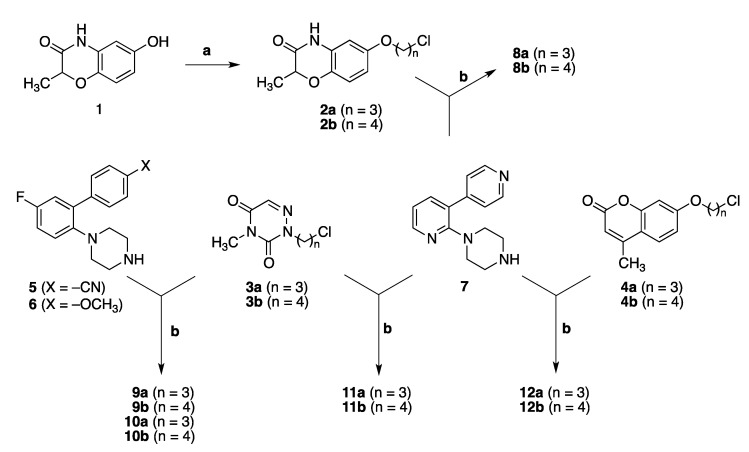
Reagents and conditions: (**a**) 1-bromo-3-chloropropane or 1-bromo-4-chlorobutane, NaH, anhydrous DMF, r.t., 12h; (**b**) Na_2_CO_3_, acetonitrile, reflux, overnight.

The synthesis of the target compounds **8a**,**b**–**12a**,**b** was accomplished through the reaction of the appropriate chloroalkyl derivatives **2a**,**b**–**4a**,**b** with arylpiperazines **5**–**7** (Scheme 1). Both chloroalkyl and arylpiperazine intermediates were synthesized with literature methods (as detailed in the Materials and Methods) except alkyl chloride **2b** which was prepared by alkylating 6-hydroxy-2-methyl-2*H*-benzo[b][1,4]oxazin-3(4*H*)-one (**1**) [16] with 1-bromo-4-chlorobutane via the Williamson’s synthesis under basic conditions.

### 2.3. Radioligand Binding Experiments

The final compounds were tested for their affinity at serotonin 5-HT_1A_, 5-HT_2A_, and 5-HT_7_ receptors and at dopamine D_1_, D_2_, D_3_, and D_5_ receptors. As a first step, competition radioligand binding assays were carried out for all the compounds at the single concentration of 10 µM, on membrane preparations from stable cell lines heterologously expressing the human cloned 5-HT_1A_, 5-HT_2A_, or 5-HT_7_ receptors. Affinity was determined for those compounds showing % of displacement of the specific binding of the radioligand over 65% at 10 µM concentration, by means of competition binding curves of six concentration datapoints (typically from 0.1 nM/1 nM to 10 µM/100 µM). The affinity of the compounds, expressed as equilibrium dissociation constant (*K*_i_) value, is shown in Table 2. For the affinity determination at dopamine receptor subtypes, membrane fractions of human dopamine D_2s_, D_3_, D_1_, and D_5_ receptor-expressing cells were used. They were co-incubated with [^3^H]spiperone (for D_2s_ and D_3_ receptor) or [^3^H]SCH23390 (for D_1_ and D_5_ receptor) and appropriate concentrations of test compound for 120 min. Bound radioligand was separated from the free ligand using filtration. Radioactivity was measured by liquid scintillation counting. The resulting *K*_i_ values with 95% confidence intervals are listed in Table 2.

### 2.4. Docking Studies

**Figure 3:**
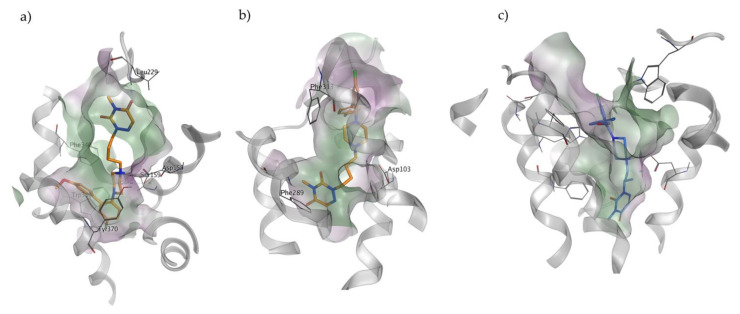
Docking poses of compound **9b** at (**a**) serotonin 5-HT_2A_ receptor; (**b**) dopamine D_1_ receptor; (**c**) dopamine D_2_ receptor.

**Figure 4:**
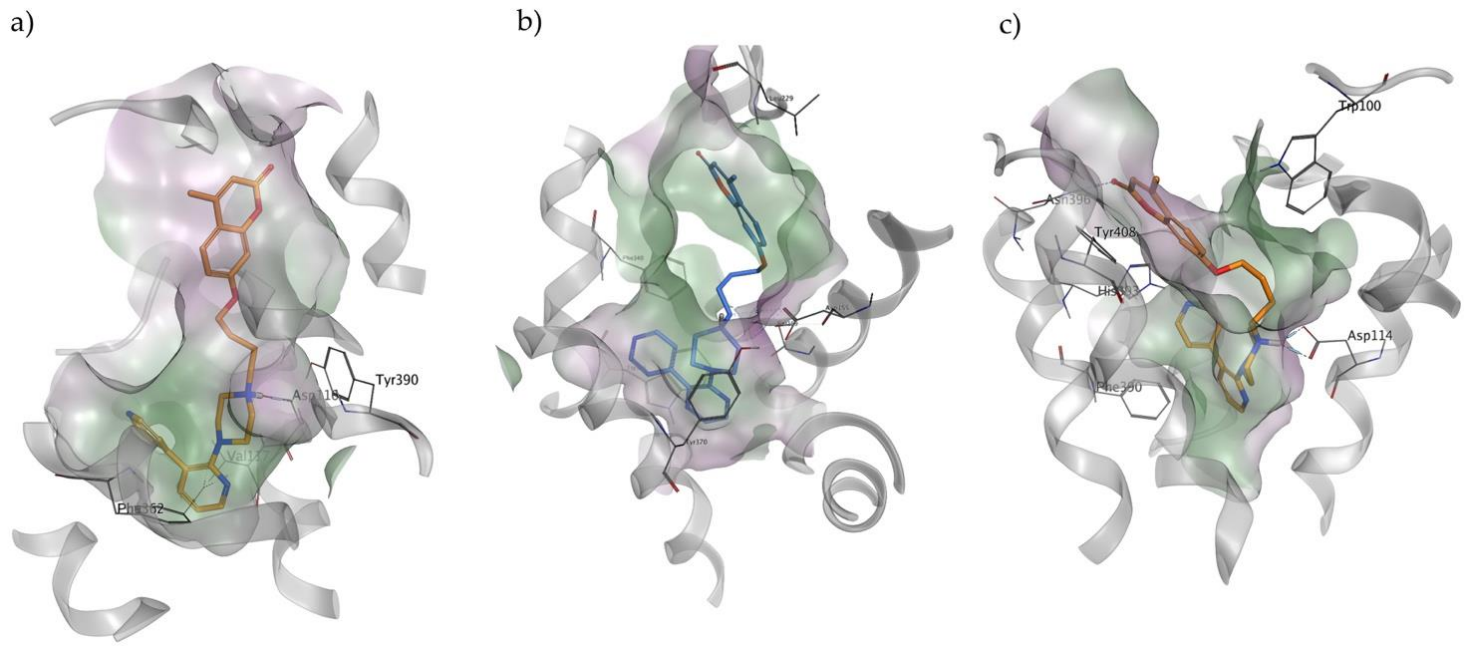
Docking poses of compound **12b** at (**a**) serotonin 5-HT_1A_ receptor; (**b**) serotonin 5-HT_2A_ receptor; (**c**) dopamine D_2_ receptor.

Docking studies were performed to rationalize the different binding profiles of the compounds. Representative compounds were docked at the serotonin 5-HT_1A_ and 5-HT_2A_, and dopamine D_2_ and D1 using the available crystal structures of the receptors (Figure 3 and Figure 4).

### 2.5. In Vitro Metabolic Stability

To evaluate the susceptibility to first-pass oxidative metabolism, the leading cause of metabolic degradation in vivo [17] the target compounds were incubated with rat liver microsomes. The turnover of the parent compound was assessed as the percentage of the parent compound recovered after 30 min of incubation with microsomes in the presence of an NADPH-regenerating system [18]. The percentages of the recovered unchanged parent compound are listed in Table 1.

### 2.6. Neuroprotection against H_2_O_2_ in SHSY-5Y Cell Line

Initially, the cytotoxicity of the target compounds was assessed by treating SH-SY5Y cells for 48 h with various concentrations of compounds (0.1 to 100 μM). Compounds **11a** and **11b** were not cytotoxic at the tested doses, while the remaining were cytotoxic, exhibiting EC_50_ values between 20–50 μM (Table 2). Cells treatment with 1 μM or 5 μM of each compound for 24 h did not result in an effect on cell viability (<4% and <8% decrease at a concentration of 1 μM and 5 μM, respectively).

**Figure 5:**
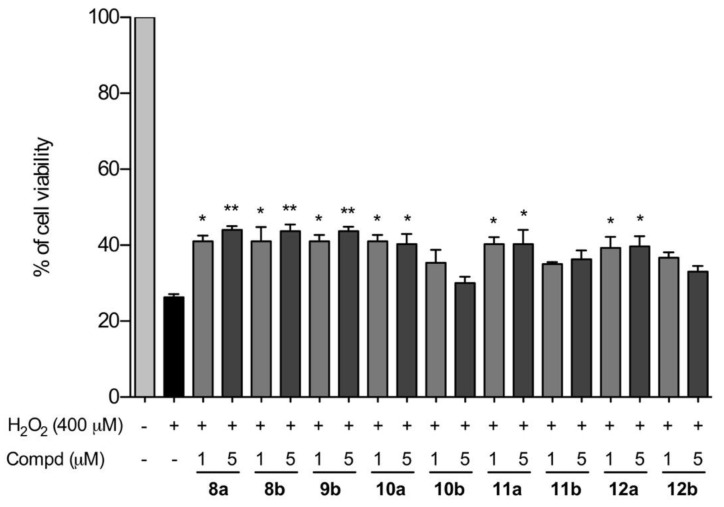
Cell viability of SH-SY5Y cells pretreated with 1 μM or 5 μM of the test compound for 3 h, and subsequently exposed to 400 μM H_2_O_2_ for 24 h. The data are shown as the mean ± S.E.M. of three independent experiments (** *p* < 0.001 * *p* < 0.05 vs. 400 μM H_2_O_2_-treated group).

Treatment of SH-SY5Y cells with 400 μM H_2_O_2_ for 24 h led to a reduction of cell viability of 72–76% (Figure 3). The protective effect of each compound was determined by pretreating the cells with the test compound for 3 h at the non-toxic doses of 1 μM and 5 μM, and subsequent treatment with 400 μM H_2_O_2_ for an additional 24 h. Pre-treatment of SH-SY5Y cells with compounds **8a**,**b**, **9b**, **10a**, **11a**, and **12a** resulted in a 14–20% recovery of cell viability with respect to treatment with the H_2_O_2_ alone (Figure 5).

## 3. Discussion

The binding affinities of the target compounds are listed in Table 2. The analysis of the binding data at the screened receptors provides information regarding the role of the three structural elements that compose the structure, i.e., biphenyl-like system, spacer, and terminal fragment.

Considering the affinities for the 5-HT_1A_ receptor, we can note that both biphenyl-like systems and terminal fragments selected for the study are compatible with high affinity at the receptor. In fact, four compounds out of 10 show *K*_i_ values lower than 17.6 nM (compounds **8a**,**b**, **11b**, **12b**). The differences in affinity are related to the length of the spacer between the piperazine and the terminal fragment. Examining the compounds characterized by the oxotriazinyl terminal fragment (compounds **9a**,**b**–**11a**,**b**), the derivatives with a tetramethylene spacer have higher affinity than the trimethylene counterparts. The influence of the spacer length on affinity is related to the nature of the terminal fragment. In fact, considering the compounds featuring the bipyridyl system linked to the piperazine, the *K*_i_ ratios of the trimethylene and tetramethylene homologs are variable (**11a**/**11b** = 52; **12a**/**12b** = 3.7; **8a**/**8b** = 0.7).

As for the 5-HT_2A_ receptor, the compounds featuring the bipyridyl system linked to the piperazine display moderate to low affinity. Within this group of compounds, the terminal fragment plays a role on affinity. In fact, the compounds with the oxotriazinyl fragment have a low affinity, whereas a bicyclic terminal fragment imparts comparatively higher affinity. The replacement of the bipyridyl with a biphenyl system has a favorable effect on the affinity. In fact, for the compounds with a tetramethylene spacer, **9b** has a slightly higher affinity than **10b** which shows much higher affinity than **11b**. A similar trend can be observed for the trimethylene spacer derivatives **9a**, **10a**, and **11a**.

The affinity values of the target compounds at 5-HT_7_ receptor are distributed in a narrower range as compared to the other receptors under study. In fact, the highest affinity values were shown by **8b** (*K*_i_ = 9.38 nM) and the lowest by **11b** (*K*_i_ = 79.4 nM). Consequently, the differences in affinity between homologs (trimethylene or tetramethylene spacer derivatives) or analogs (i.e., pair of compounds featuring the same biphenyl-like system and a different terminal fragment) are always lower than 2-fold.

As for the D_2_-like receptors (D_2_ and D_3_), it is immediately evident that the oxotriazinyl terminal fragment leads to compounds devoid of affinity (compounds **9a**,**b**–**11a**,**b**). By contrast, the bicyclic terminal fragments lead from moderate to high affinity (compounds **8a**,**b**, **12a**,**b**) to both D_2_ and D_3_ receptors, being the affinity for the former receptor slightly higher. For these compounds, the tetramethylene derivatives have slightly higher affinity than the trimethylene analogs.

Regarding the D_1_-like receptors (D_1_ and D_5_), the biphenyl-like system influences the affinity to a greater extent than the terminal fragment. In fact, the bipyridyl fragment leads to low affinity for D_1_ and D_5_ receptors, whereas the biphenyl fragment to moderate affinity. Considering compounds **9a**,**b** and **10a**,**b**, the trimethylene or tetramethylene homologs display a difference in affinity lower than 2-fold.

Collectively, the binding data of the target compounds revealed that the three structural elements that compose the structure have different effects on the affinity for the receptors under study. For 5-HT_1A_ receptor, variations of length of the spacer result in the highest variations in affinity. For 5-HT_2A_ receptors, the nature of the biphenyl-like system has high influence on the affinity. For 5-HT_7_ receptor, the biphenyl-like systems linked to the piperazine ring confirmed their favorable role on affinity, with no substantial effects of both the spacer length and the terminal fragments. For D_2_-like receptor, the terminal fragment has a pronounced impact on affinity, whereas for D_1_-like receptor biphenyl-like system has a greater effect.

To identify molecular contacts responsible for the observed affinity profile, compounds **9b** and **12b** were docked at serotonin 5-HT_1A_ and 5-HT_2A_ and dopamine D_2_ and D_1_ receptors. We found that compounds orientations were analogous for all the studied receptors, except for the dopamine D_1_ receptor, with the piperazine moiety forming a strong charged-assisted hydrogen bond with the highly conserved aspartate residue on the third transmembrane helix and the aryl substituent linked to the piperazine ring deeply buried in the binding site. By comparing the docking poses at serotonin 5-HT_2A_ receptor of compounds **9b** (5-HT_2A_ *K*_i_ = 39.4 nM) and **12b** (5-HT_2A_ *K*_i_ = 1492 nM), it is evident that the dimension of the terminal fragment impacts the interaction of the molecule with the receptor. In fact, the oxotriazinyl terminal fragment of compound **9b** formed an additional contact with Leu229 of ECL2. In contrast, the bicyclic terminal fragment of compound **12b** is too big to allow a proper accommodation in the binding pocket and leads to a steric clash. Similarly, compounds **9b** and **12b** showed very different binding poses at the dopamine D_2_ receptor, which agree with the very different *K*_i_ values (compound **9b** *K*_i_ = >10,000 nM, compound **12b** *K*_i_ = 17.0 nM). In fact, while the arylpiperazine moiety of compound **12b** is located deeper into the binding site and the terminal fragment is orientated towards the extracellular milieu forming an additional contact with Asn396, compound **9b** showed an opposite orientation with the arylpiperazine pointing towards the extracellular milieu that is not in agreement with the observed structure–activity relationships.

Binding affinity data and docking poses indicated that, when the structural requirements for high affinity for a given set of receptors overlap, compounds with different profiles can be identified. In fact, compounds **9b** and **10b** have a balanced profile concerning the affinity for 5-HT_1A_, 5-HT_2A_, and 5-HT_7_ receptors, which is the desired affinity profile to obtain prospective drugs for the treatment of the core symptoms of ASD. In addition, the selectivity over D_2_ receptors of both **9b** and **10b** is indeed favorable in terms of potential extrapyramidal side effects. On the other hand, compounds **8b** and **12a** have an affinity profile compatible with antipsychotic activity, as they have an affinity for the D_2_ receptor, being **12a** preferable for the balanced affinity for D_2_ and 5-HT_2A_ receptors, accompanied by a comparable affinity for 5-HT_1A_ and 5-HT_7_ receptors.

Next, we studied the potential of the target compounds to protect against apoptosis induced by H_2_O_2_ using SH-SY5Y neuroblastoma cells as an experimental model.

We first evaluated the cytotoxicity of the target compounds alone and found that compounds **11a** and **11b** were not cytotoxic in the dose range of 0.1 to 100 μM, while the remaining compounds showed moderate cytotoxicity, having EC_50_ values between 20–50 μM (Table 2). Of note, SH-SY5Y cells treatment with each compound at 1 μM or 5 μM concentration for 24 h had a marginal effect on cell viability, with a decrease lower than 4% or 8% at 1 μM or 5 μM, respectively.

Next, the protective effect of each compound was determined by pretreating the cells with the test compound for 3 h at the non-toxic doses of 1 μM and 5 μM, and subsequent treatment with 400 μM H_2_O_2_ for an additional 24 h. Treatment of SH-SY5Y cells with 400 μM H_2_O_2_ for 24 h caused a reduction of cell viability of 72–76% (Figure 3). Pretreatment of SH-SY5Y cells with compounds **8a**,**b**, **9b**, and **10a**–**12a** resulted in a statistically significant recovery of cell viability with respect to treatment with the 400 μM H_2_O_2_ alone at both concentrations studied (Figure 3). Thus, compounds **8b**, **9b**, and **12a** stand out of the set because they combine a favorable affinity profile with antioxidant properties.

The target compounds were screened for their in vitro metabolic stability to evaluate their liability to metabolic degradation by first-pass oxidative metabolism—the main cause of metabolic degradation in vivo [17]. The metabolic stability was assessed as the percentage of the parent compound recovered after 30 min of incubation with rat microsomes in the presence of an NADPH-regenerating system (Table 1). Compound **9b** was the most stable compound as it showed a percentage of recovery (45%) that is predictive of low clearance in vivo, based on our previous data on a broad set of analog arylpiperazine derivatives [5,18]. Among the remaining compounds, **10b** and **12a** showed borderline stability value, while the others were massively metabolized. By comparing the cLogP values and metabolic stability of each compound, it emerges that the compound’s oxidative liability is not related to lipophilicity, as shown by pairs of compounds **9a**,**b** and **10a**,**b** (Table 1).

Finally, the solubility of compounds **9b** and **12a**, which showed the best combination of biological activity and metabolic stability, were calculated using the Swiss ADME algorithm [19]. Both compounds were predicted as moderately soluble showing solubility values in the same range of buspirone and aripiprazole (solubility **9b**: 5.08 × 10^−3^ mg/mL; **12a**: 5.06 × 10^−3^ mg/mL; buspirone: 8.64 × 10^−2^ mg/mL; aripiprazole: 1.88 × 10^−3^ mg/mL).

## 4. Materials and Methods

### 4.1. Chemicals

Chemicals were purchased from Sigma-Aldrich, Alfa Aesar, TCI Chemicals. Unless otherwise stated, all chemicals were used without further purification. Thin layer chromatography (TLC) was performed using plates from Merck (silica gel 60 F254). Column chromatography was performed with 1:30 Merck silica gel 60 Å (63–200 µm) as the stationary phase. Flash chromatographic separations were performed on a Biotage SP1 purification system using flash cartridges pre-packed with KP-Sil 32−63 μm, 60 Å silica. ^1^H NMR spectra were recorded on a Varian Mercury-VX spectrometer (300 MHz) or on a 500-vnmrs500 Agilent spectrometer (500 MHz). All chemical shift values are reported in ppm (δ). Recording of mass spectra was done on an HP6890-5973 MSD gas chromatograph/mass spectrometer; only significant *m/z* peaks, with their percentage of relative intensity in parentheses, are reported. HRMS-ESI analyses were performed on a Bruker Datonics MicrOTOF-Q II mass spectrometer, mass range 50–800 *m/z*, electrospray ion source in positive or negative ion mode. All spectra were in accordance with the assigned structures. Elemental analyses (C,H,N) of the target compounds were performed on a Eurovector Euro EA 3000 analyzer. Analyses indicated by the symbols of the elements were within ± 0.4% of the theoretical values. The purity of the target compounds listed in Table 1 was assessed by RP-HPLC and combustion analysis. All compounds showed ≥95% purity. RP-HPLC analysis was performed on an Agilent 1260 Infinity Binary LC System equipped with a diode array detector using a Phenomenex Gemini C-18 column (250 mm × 4.6 mm, 5 μm particle size). All target compounds (Table 1) were eluted with CH_3_CN/ammonium formate 50 mM pH 5, 8:2 (*v*/*v*) at a flow rate of 1 mL/min.

The following compounds were prepared as described in the literature: 6-hydroxy-2-methyl-2*H*-benzo[b][1,4]oxazin-3(4*H*)-one (**1**) [20], 6-(3-chloropropoxy)-2-methyl-2*H*-benzo[b][1,4]oxazin-3(4*H*)-one (**2a**) [5], 2-(3-chloropropyl)-4-methyl-1,2,4-triazine-3,5(2*H*,4*H*)-dione (**3a**) [21], 2-(4-chlorobutyl)-4-methyl-1,2,4-triazine-3,5(2*H*,4*H*)-dione (**3b**) [21], 7-(3-chloropropoxy)-4-methyl-2*H*-chromen-2-one (**4a**) [5], 7-(4-chlorobutoxy)-4-methyl-2*H*-chromen-2-one (**4b**). [5], 5’-fluoro-2’-(piperazin-1-yl)-[1,1’-biphenyl]-4-carbonitrile (**5**) [18], 1-(5-fluoro-4’-methoxy-[1,1’-biphenyl]-2-yl)piperazine (**6**) [18], 2-(piperazin-1-yl)-3,4’-bipyridine (**7**) [22].

### 4.2. Synthesis

*6-(4-Chlorobutoxy)-2-methyl-2H-benzo[b][1,4]oxazin-3(4H)-one (***2b***).* To a suspension of NaH (0.11 g, 4.47 mmol) in anhydrous DMF (3 mL), 6-hydroxy-2-methyl-2*H*-benzo[b][1,4]oxazin-3(4*H*)-one (**1**) (0.61 g, 3.43 mmol) was added portionwise. The mixture was stirred at room temperature for 10 min, then a solution of the appropriate 1-bromo-4-chlorobutane (0.70 g, 4.47 mmol) in the same solvent (2 mL) was added. The reaction mixture was stirred at room temperature for 12 h, poured on ice water and extracted with AcOEt (3 × 20 mL). The organic layers were collected, washed with brine, dried over Na_2_SO_4_ and concentrated under reduced pressure. The crude was purified on a silica gel column by gradient elution from *n*-hexane/AcOEt 7:3 to *n*-hexane/AcOEt, 1:1, to obtain the pure compound as a yellow solid (0.31 g, 36% yield). ^1^H NMR (CDCl_3_): δ 1.56 (d, 3H, *J* = 6.8 Hz), 1.95 (qn, 2H, *J* = 6.0 Hz), 2.21 (qn, 2H, *J* = 6.0 Hz), 3.43 (t, 2H, *J* = 6.4 Hz), 3.73 (t, 2H, *J* = 6.4 Hz), 4.59 (q, 1H, *J* = 6.8 Hz), 6.37 (d, 1H, *J* = 2.5 Hz), 6.52 (dd, 1H, *J* = 2.9 and 8.8 Hz), 6.89 (d, 1H, *J* = 8.8 Hz), 8.14 (s, 1H, D_2_O exchanged). GC/MS *m/z* 271 (M^+^+2, 20), 269 (M^+^, 60), 179 (100), 1336 (65), 55 (50).

*General Procedure for the Preparation of the Final Compounds ***8a**,**b**–**12a**,**b****

A stirred mixture of the alkylating agent **2a**,**b**–**4a**,**b** (0.9 mmol), the piperazine **6**–**7** (1.08 mmol) and K_2_CO_3_ (0.15 g, 1.08 mmol) in acetonitrile (20 mL) was refluxed overnight. After cooling, the mixture was evaporated to dryness and H_2_O (20 mL) was added to the residue. The aqueous phase was extracted with AcOEt (3 × 20 mL). The collected organic layers were dried over Na_2_SO_4_ and evaporated under reduced pressure. The crude residue was purified by chromatographic column using CHCl_3_/MeOH, 19:1 to give the pure target compound.

*6-(3-(4-([3,4’-Bipyridin]-2-yl)piperazin-1-yl)propoxy)-2-methyl-2H-benzo[b][1,4]oxazin-3(4H)-one (***8a***).* Yellow solid, 41% yield. ^1^H NMR (CDCl_3_): δ 1.55 (d, 3H, *J* = 6.4 Hz), 1.89–1.93 (m, 2H), 2.42 (br s, 4H), 2.48–2.53 (m, 2H), 3.13 (br s, 4H), 3.92 (t, 2H, *J* = 6.4 Hz), 4.53–4.60 (m, 1H), 6.33 (d, 1H, *J* = 2.3 Hz), 6.47 (dd, 1H, *J* = 2.3 Hz and 8.8 Hz), 6.86 (d, 1H, *J* = 8.8 Hz), 6.95 (dd, 1H, *J* = 4.7 Hz and 7.6 Hz), 7.47 (dd, 1H, *J* = 1.8 Hz and 7.6 Hz), 7.55 (d, 2H, *J* = 5.9 Hz), 8.17 (br s, 1H), 8.27 (dd, 1H, *J* = 1.8 Hz and 4.7 Hz), 8.65 (d, 2H, *J* = 4.68 Hz). HRMS (ESI^+^) calcd for [(C_26_H_29_N_5_O_3_)+Na]^+^: 482.2163, found: 482.2169. ESI^+^/MS *m/z* 482 (M^+^). ESI^+^/MS/MS *m/z* 482 (100); Mp 179–180 °C (from CHCl_3_/*n*-hexane).

*6-(4-(4-([3,4’-Bipyridin]-2-yl)piperazin-1-yl)butoxy)-2-methyl-2H-benzo[b][1,4]oxazin-3(4H)-one (***8b***).* Yellow solid, 33% yield. ^1^H NMR (CDCl_3_): δ 1.55 (d, 3H, *J* = 7.0 Hz), 1.60–1.67 (m, 2H), 1.71–1.80 (m, 2H), 2.36–2.41 (m, 6H), 3.11–3.14 (m, 4H), 3.89 (t, 2H, *J* = 6.4 Hz), 4.57 (q, 1H, *J* = 6.4 Hz), 6.32 (d, 1H, *J* = 2.3 Hz), 6.47 (dd, 1H, *J* = 2.9 Hz and 8.8 Hz), 6.87 (d, 1H, *J* = 8.8 Hz), 6.95 (dd, 1H, *J* = 7.0 Hz and 7.61 Hz), 7.47 (dd, 1H, *J* = 1.8 Hz and 7.6 Hz), 7.54–7.57 (m, 2H), 8.11 (br s, 1H), 8.27 (dd, 1H, *J* = 1.8 Hz and 4.7 Hz), 8.65 (d, 2H, *J* = 5.9 Hz). HRMS (ESI^+^) calcd for [(C_27_H_31_N_5_O_3_)+Na]^+^: 496.2319, found: 496.2319. ESI*^+^*/MS *m*/*z* 496 (M+Na)^+^. ESI^-^/MS/MS *m/z* 496 (100), 295 (66), 226 (56). Mp 174–175 °C (from CHCl_3_/*n*-hexane).

*2-(3-(4-(5-Fluoro-4’-methoxy-[1,1’-biphenyl]-2-yl)piperazin-1-yl)propyl)-4-methyl-1,2,4-triazine-3,5(2H,4H)-dione (***9a***).* Yellow oil, 17% yield. ^1^H NMR (CDCl_3_): δ 1.68–1.80 (m, 2H), 2.31–2.35 (m, 6H), 2.77–2.80 (m, 4H), 3.32 (s, 3H), 3.82 (s, 3H), 3.97 (t, 2H, *J* = 7.0 Hz), 6.90–6.98 (m, 5H), 7.37 (s, 1H), 7.54–7.56 (m, 2H). HRMS (ESI^+^) calcd for [(C_24_H_28_FN_5_O_3_)+Na]^+^: 476.2068, found: 476.2068. ESI^+^/MS *m/z* 476 (M+Na)^+^. ESI^+^/MS/MS *m/z* 476 (100).

*2-(4-(4-(5-Fluoro-4’-methoxy-[1,1’-biphenyl]-2-yl)piperazin-1-yl)butyl)-4-methyl-1,2,4-triazine-3,5(2H,4H)-dione (***9b***).* Dark brown oil, 24% yield. ^1^H NMR (CDCl_3_): δ 1.49–1.54 (m, 2H), 1.67–1.80 (m, 2H), 2.31–2.36 (m, 6H), 2.77–2.80 (m, 4H), 3.32 (s, 3H), 3.85 (s, 3H), 3.97 (t, 2H, *J* = 7.0 Hz), 6.90–6.98 (m, 5H), 7.37 (s, 1H), 7.54–7.56 (m, 2H). HRMS (ESI^+^) calcd for [(C_25_H_30_FN_5_O_3_)+Na]^+^: 490.2225, found: 490.2210. ESI^+^/MS *m/z* 490 (M+Na)^+^. ESI^+^/MS/MS *m/z* 341 (14).

*5’-Fluoro-2’-(4-(3-(4-methyl-3,5-dioxo-4,5-dihydro-1,2,4-triazin-2(3H)-yl)propyl)piperazin-1-yl)-[1,1’-biphenyl]-4-carbonitrile (***10a***).* Yellow oil, 51% yield. ^1^H NMR (CDCl_3_): δ 1.83–1.93 (m, 2H), 2.29 (br s, 4H), 2.36–2.40 (m, 2H), 2.71(app t, 4H), 3.31 (s, 3H), 4.02 (t, 2H, *J* = 7.0 Hz), 6.92 6.95 (m, 1H), 7.00–7.06 (m, 2H), 7.36 (s, 1H), 7.66–7.76 (m, 4H). HRMS (ESI^+^) calcd for [(C_24_H_25_FN_6_O_2_)+Na]^+^: 471.1915, found: 471.1888. ESI^+^/MS *m/z* 471 (M+Na)^+^. ESI^+^/MS/MS *m/z* 471 (100), 316 (53).

*5’-Fluoro-2’-(4-(4-(4-methyl-3,5-dioxo-4,5-dihydro-1,2,4-triazin-2(3H)-yl)butyl)piperazin-1-yl)-[1,1’-biphenyl]-4-carbonitrile (***10b***).* Brown oil, 48% yield. ^1^H NMR (CDCl_3_): δ 1.44–1.53 (m, 2H), 1.71–1.81 (m, 2H), 2.30–2.35 (m, 6H), 2.75–2.78 (m, 4H), 3.31 (s, 3H), 3.97 (t, 2H, *J* = 7.0 Hz), 6.92 6.98 (m, 1H), 7.00–7.06 (m, 2H), 7.37 (s, 1H), 7.66–7.74 (m, 4H). HRMS (ESI^+^) calcd for [(C_25_H_27_FN_6_O_2_)+Na]^+^: 485.2072, found: 485.2058. ESI^+^/MS *m/z* 485 (M+Na)^+^. ESI^+^/MS/MS *m/z* 336 (100).

*2-(3-(4-([3,4’-Bipyridin]-2-yl)piperazin-1-yl)propyl)-4-methyl-1,2,4-triazine-3,5(2H,4H)-dione (***11a***).* Yellow oil, 29% yield. ^1^H NMR (CDCl_3_): δ 1.84–1.92 (m, 2H), 2.34–2.38 (m, 4H), 2.40 (t, 2H, *J* = 7.0 Hz), 3.06 (app t, 4H), 3.31 (s, 3H), 4.03 (t, 2H, *J* = 6.4 Hz), 6.93 (dd, 1H, *J* = 7.0 Hz and 7.6 Hz), 7.35 (s, 1H), 7.46 (dd, 1H, *J* = 1.8 and 7.6), 7.51 (dd, 2H, *J* = 1.8 Hz and 4.7 Hz), 8.25 (dd, 1H, *J* = 1.8 Hz and 4.7 Hz ), 8.63 (d, 2H, *J* = 5.9 Hz). HRMS (ESI^+^) calcd for [(C_21_H_25_N_7_O_3_)+Na]^+^: 430.1962, found: 430.1960. ESI^+^/MS *m/z* 430 (M+Na)^+^. ESI^+^/MS/MS *m/z* 430 (100), 64 (26).

*2-(4-(4-([3,4’-Bipyridin]-2-yl)piperazin-1-yl)butyl)-4-methyl-1,2,4-triazine-3,5(2H,4H)-dione (***11b***).* Brown solid, 10% yield. ^1^H NMR (CDCl_3_): δ 1.50–1.52 (m, 2H), 1.70–1.78 (m, 2H), 2.33–2.38 (m, 6H), 3.30–3.32 (m, 4H), 3.31 (s, 3H), 3.97 (t, 2H, *J* = 7.0 Hz), 6.93 (dd, 1H, *J* = 7.0 Hz and 7.6 Hz), 7.37 (s, 1H), 7.45–7.48 (m, 1H), 7.51–7.56 (m, 2H), 8.26–8.29 (m, 1H), 8.63–8.68 (m, 2H). HRMS (ESI^+^) calcd for [(C_22_H_27_N_7_O_2_)+Na]^+^: 444.2118, found: 444.2111. ESI^+^/MS *m/z* 444 (M+Na)^+^. ESI^+^/MS/MS *m/z* 444 (100), 226 (46). Mp 165–167 °C (from CHCl_3_/*n*-hexane).

*7-(3-(4-([3,4’-Bipyridin]-2-yl)piperazin-1-yl)propoxy)-4-methyl-2H-chromen-2-one (***12a***).* Yellow oil, 41% yield. ^1^H NMR (CDCl_3_): δ 1.92–2.01 (m, 2H), 2.38 (s, 3H), 2.39 (app t, 4H), 2.51 (t, 2H, *J* = 7.61 Hz), 3.13 (app t, 4H), 4.06 (t, 2H, *J* = 5.9 Hz), 6.12 (s, 1H), 6.80–6.84 (m, 2H), 6.96 (dd, 1H, *J* = 5.3 Hz and 7.6 Hz), 7.45–7.48 (m, 2H), 7.53–7.6 (m, 2H), 8.26 (dd, 1H, *J* = 1.8 Hz and 4.7 Hz), 8.64–8.66 (m, 2H). HRMS (ESI^+^) calcd for [(C_27_H_28_N_4_O_3_)+Na]^+^: 479.2054, found: 479.2052. ESI^+^/MS *m/z* 479 (M+Na)^+^. ESI^+^/MS/MS *m/z* 479 (100), 281 (55), 84 (23).

*7-(4-(4-([3,4’-Bipyridin]-2-yl)piperazin-1-yl)butoxy)-4-methyl-2H-chromen-2-one (***12b***).* Yellow oil, 18% yield. ^1^H NMR (CDCl_3_): δ 1.62–1.69 (m, 2H), 1.80–1.86 (m, 2H), 2.39 (s, 3H), 2.40–2.43 (m, 4H), 3.13 (t, 2H, *J* = 5.4 Hz), 3.14–3.19 (m, 4H), 4.02 (t, 2H, *J* = 6.9 Hz), 6.12 (s, 1H), 6.78 (d, 1H, *J* = 2.4 Hz), 6.83 (dd, 1H, *J* = 2.4 Hz and 8.8 Hz), 6.93 (dd, 1H, *J* = 4.9 Hz and 7.3 Hz), 7.46–7.48 (m, 2H), 7.53–7.55 (m, 2H), 8.28 (dd, 1H, *J* = 4.9 Hz and 9.3 Hz), 8.65 (d, 1H, *J* = 5.9 Hz), 8.67 (d, 1H, *J* = 5.9 Hz). HRMS (ESI^+^) calcd for [(C_28_H_30_N_4_O_3_)+Na]^+^: 493.2210, found: 493.2214. ESI^+^/MS *m/z* 493 (M+Na)^+^. ESI^+^/MS/MS *m/z* 295 (100), 226 (90).

### 4.3. Radioligand Binding Assays

#### 4.3.1. 5-HT_1A_ Receptor

The affinity of the compounds for serotonin 5-HT_1A_ receptor was evaluated in membrane preparations from HEK293 cells stably expressing the human cloned receptor, following previously described procedures [23] with minor modifications. Competition binding experiments were performed using 1 nM [^3^H]-8-Hydroxy-DPAT (1 mCi/mL, PerkinElmer NET929250UC) as radioligand. Nonspecific binding was assessed in the presence of 10 µM serotonin. 5-Carboxamidotryptamine (5-CT) was included in the assays as reference compound. Assays were carried out in duplicate in 96-well assay plates, in assay buffer (50 mM Tris-HCl, 5 mM MgSO_4_, pH = 7.4). Assay mixtures were incubated at 37 °C for 120 min, followed by filtration through GF/C glass filter plates, and washed with ice-cold wash buffer (50 mM Tris-HCl, pH = 7.4). Competition binding curves, typically constructed with six different concentrations of the compounds, were fitted to a one-site competition model using Prism 6 software (GraphPad, San Diego, CA, USA), and equilibrium dissociation constant (*K*_i_) of the compounds was calculated according to the Cheng–Prusoff equation. *K*_i_ value of the reference ligand 5-CT was 0.32 ± 0.02 nM.

#### 4.3.2. 5-HT_2A_ Receptor

The affinity of the compounds for serotonin 5-HT_2A_ receptor was evaluated in membrane preparations from CHO-K1 cells stably expressing the human cloned receptor, following previously described procedures [23] with minor modifications. Competition binding experiments were performed using 1 nM [^3^H]-ketanserin (50.3 Ci/mmol, 1 mCi/mL, PerkinElmer NET791250UC) as radioligand. Nonspecific binding was assessed in the presence of 1 µM methysergide. Risperidone was included in the assays as reference compound. Assays were carried out in duplicate in 96-well assay plates, in assay buffer (50 mM Tris-HCl, pH = 7.4). Assay mixtures were incubated at 37 °C for 30 min, followed by filtration through GF/B glass filter plates, and wash with ice-cold wash buffer (50 mM Tris-HCl, pH = 6.6). Affinity (equilibrium dissociation constant, *K*_i_) of the compounds was calculated from competition binding curves as indicated above. *K*_i_ value of the reference ligand risperidone was 0.20 ± 0.04 nM.

#### 4.3.3. 5-HT_7_ Receptor

The affinity of the compounds for serotonin 5-HT_7_ receptor was evaluated in membrane preparations from HEK293 cells stably expressing the human cloned 5-HT_7A_ receptor, following previously described procedures [24] with minor modifications. Competition binding experiments were performed using 2 nM [^3^H]-SB269970 (34.5 Ci/mmol, 0.25 mCi/mL, Perkin Elmer NET1198U250UC) as radioligand. Nonspecific binding was assessed in the presence of 25 µM clozapine. Methiothepine was included in the assays as reference compound. Assays were carried out in duplicate in 96-well assay plates, in assay buffer (50 mM Tris-HCl, 4 mM MgCl_2_, 1 mM ascorbic acid, 0.1 mM pargyline, pH = 7.4). Assay mixtures were incubated at 37 °C for 60 min, followed by filtration through GF/C glass filter plates, and washing with ice-cold assay buffer. Affinity (equilibrium dissociation constant, *K*_i_) of the compounds was calculated from competition binding curves as indicated above. *K*_i_ value of the reference ligand methiothepin was 1.33 ± 0.32 nM.

#### 4.3.4. Dopamine Receptors

Competition binding experiments at dopamine D_2s_ and D_3_ receptor were performed to determine the affinity of the compounds. Cell culture conditions are described in Frank et al. [25]. Membrane preparations of transfected CHO-K1 cells stably expressing the human D_2s_ or D_3_ receptor were performed according to Bautista-Aguilera et al. [26]. The competition binding experiments were conducted as previously reported [26,27]. Briefly, membrane fractions (D_2s_R: 25 µg/200 µL; D_3_R: 20 µg/200 µL) were incubated with [^3^H]spiperone (0.2 nM) and test compound for 120 min at room temperature. Seven appropriate concentrations of compound between 100 µM and 0.01 nM were used. Non-specific binding was determined in the presence of 10 µM haloperidol. Haloperidol was also used as reference compound. Separation of the bound ligand from the free ligand was conducted by filtration through GF/B filters using deionized water.

The affinity for dopamine D_1_ and D_5_ receptors was evaluated in membrane fractions of HEK-293 cells stably expressing the human dopamine D_1_ or D_5_ receptor. HEK-293-D_1_ cells were cultured in DMEM/F12 (15 mM HEPES, 1.2 g/L NaHCO_3_) with 10% (*v*/*v*) FBS and 1% (*v*/*v*) L-glutamine. HEK-293-D_5_ cells were cultured in DMEM/F12 (with L-glutamine, 1.2 g/L NaHCO_3_) with 20% (*v*/*v*) FBS. Preparation of membrane fractions and radioligand competition assays were performed as previously described [26]. Briefly, membrane fractions were co-incubated with [^3^H]-SCH23390 (0.3 nM) and different concentrations of test compound for 120 min at room temperature. Fluphenazine (100 µM) was used to determine non-specific binding and as reference compound.

Data of at least three independent experiments in triplicate were analyzed using GraphPad Prism 8.1 (San Diego, CA, USA). Binding curves were fitted to a non-linear regression model (one-site competition). *K*_i_ values were calculated from IC_50_ values using the Cheng–Prusoff equation. The statistical calculations were performed on –log(*K*_i_). Results are presented in mean *K*_i_ (nM) with 95% confidence intervals (nM).

### 4.4. Docking Studies

The structures of compounds **8b**, **9b**, **12a**, and **12b** were loaded into MOE 2019.0102 (Chemical Computing Group, Montreal, Canada). Subsequently, the protonation state was adjusted to dominant at pH = 7.0 and the compounds were subjected to energy minimization. X-ray structures of 5-HT_1A_ (PDB code: 7E2Z [28]), 5-HT_2A_ (PDB code: 6WHA [29]), D_1_ (PDB code: 7JOZ [30]), and D_2_ (PDB code: 6CM4 [31]) were loaded into MOE 2019.0102 and subjected to “QuickPrep” routine, which includes correction of missing loops, adjustment of protonation state and energy minimization. For docking, the initial placement was performed by setting the central interaction towards the acidic aspartate as essential. Initial scoring of 30 poses was performed using London dG scoring function, while refinement was performed with a rigid receptor, and the five best poses were selected based on GBVI/WSA dG scoring function. The best pose was subjected to energy minimization and inspected manually.

### 4.5. Stability Assays in Rat Liver Microsomes

Test compounds were pre-incubated at 37 °C with rat liver microsomes (Tebu-Bio, Milan, Italy) (1.0 mg/mL microsomal protein) at 10 μM final concentration in 100 mM potassium phosphate buffer (pH 7.4) for 10 min. Metabolic reactions were initiated by the addition of the NADPH regenerating system (containing 10 mM NADP, 50 mM glucose-6-phosphate, and 10 unit/mL glucose-6-phosphate dehydrogenase, final glucose-6-phosphate dehydrogenase concentration, 1 unit/mL). After 30 min incubation, the reaction mixture was quenched by adding an equal volume of cold acetonitrile containing the internal standard. A test compound incubated with microsomes without NADPH regenerating system was included. Quenched samples were centrifuged at 4500 rpm for 15 min and the supernatants were injected for quantification analysis. Samples (100 μL) were analyzed by using an Agilent 1260 Infinity Binary LC System equipped with a diode array detector (Open Lab software was used to analyze the chromatographic data) and a Phenomenex Gemini C-18 column (250 mm × 4.6 mm, 5 μm particle size). The samples were eluted using CH_3_CN/20 mM ammonium formate pH 5.5 (7:3, *v*/*v*) as eluent (volumetric flow rate =1 mL/min). Concentrations were quantified by measuring the area under the peak.

The percentage of the parent compound remaining after a 30-min incubation was calculated according to the equation:

% of parent compound remaining after 30 min = C_parent_/C_control_ × 100 (1)

where C_parent_ is ligand concentration after incubation with microsome fraction and NADPH regenerating system and C_control_ is ligand concentration after incubation with microsome fraction only.

### 4.6. Evaluation of Cell Viability

#### 4.6.1. Cell Culture

SH-SY5Y neuroblastoma cells (cat. CRL-2266, ATCC) were cultured in a 1:1 mixture of Eagle’s Minimum Essential Medium (cat. 15-010-CVR, Corning) and Ham’s F12 medium (cat. 10-080-CVR, Corning). This medium was supplemented with 10% (*v*/*v*) Fetal Bovine Serum (cat. 35-079-CV, Corning), 1% (*v*/*v*) Glutamine (cat. ECB3000D, Euro Clone) and 1% (*v*/*v*) Penicillin—Streptomycin (cat.30-002-CI, Corning). Cells were cultivated at 37 °C with 5% CO_2_ at saturated humidity.

#### 4.6.2. Cell Viability

Determination of cell growth was performed using the MTT assay at 48 h. [32] On day 1, 25,000 cells/well were seeded into 96-well plates in a volume of 100 μL. On day 2, the various drug concentrations (1 μM–100 μM) were added. In all the experiments, the various drug-solvents (EtOH, DMSO) were added in each control to evaluate possible solvent cytotoxicity. After the established incubation time with drugs (48 h), MTT (0.5 mg/mL) was added to each well, and after 3–4 h incubation at 37 °C, the supernatant was removed. The formazan crystals were solubilized using 100 μL of DMSO/EtOH (1:1, *v*/*v*) and the absorbance values at 570 and 630 nm were determined on the microplate reader Victor 3 (PerkinElmer Life Sciences, Waltham, MA, USA).

#### 4.6.3. Evaluation of Cell Viability

Determination of neuroprotection against H_2_O_2_ was performed using the MTT assay at 24 h. [33] On day 1, 25,000 cells/well were seeded into 96-well plates in a volume of 100 μL. On day 2, the cells were pretreated (3 h) with two drug concentrations (1 μM and 5 μM) before being incubated with 400 μM H_2_O_2_ for 24 h. In all the experiments, the various drug-solvents (EtOH, DMSO) were added in each control to evaluate a possible solvent cytotoxicity. After 24 h incubation, MTT (0.5 mg/mL) was added to each well, and after 3–4 h incubation at 37 °C, the supernatant was removed. The formazan crystals were solubilized using 100 μL of DMSO/EtOH (1:1, *v*/*v*) and the absorbance values at 570 and 630 nm were determined on the microplate reader Victor 3 from PerkinElmer Life Sciences (Waltham, MA, USA).

#### 4.6.4. Statistical Analysis

Data were analyzed by applying the one-way repeated measures analysis of variance, and Bonferroni’s multiple comparison test followed as a post hoc test. Results are reported as mean SD of at least two to three independent experiments, performed in triplicate. Statistical significance was accepted at *p* < 0.05.

## 5. Conclusions

In the present study, we have described the design, synthesis, and biological evaluation of a set of long-chain arylpiperazine derivatives. The structural modification led to the identification of new compounds displaying an array of affinity for serotonin 5-HT_1A_, 5-HT_2A_, 5-HT_7_ receptors, and dopamine D_2_ receptor and, in some cases, antioxidant properties. Binding affinity data evidenced that: (i) the nature of the terminal fragment had an impact mostly on the affinity at dopamine D_1_- and D_2_-like receptors; (ii) the length of the linker influenced the affinity at serotonin 5-HT_1A_ receptor; (iii) the nature of the biphenyl-like system linked to the piperazine ring influenced the affinity at serotonin 5-HT_2A_ and 5-HT_7_ receptors. The most interesting compounds were: **12a** that combines an affinity profile compatible with antipsychotic activity (affinity for dopamine D_2_ (*K*_i_ = 300 nM) and 5-HT_2A_ (*K*_i_ = 315 nM) receptors, accompanied by an affinity for 5-HT_1A_ (*K*_i_ = 41.5 nM) and 5-HT_7_ (*K*_i_ = 42.5 nM) receptors) and antioxidant properties; **9b** that has an affinity profile compatible for studies in the context of ASD (affinity for serotonin 5-HT_1A_ (*K*_i_ = 23.9 nM), 5-HT_2A_, (*K*_i_ = 39.4 nM), 5-HT_7_ (*K*_i_ = 45.0 nM) receptors and selectivity over dopamine D_2_ receptors) and antioxidant properties. Even though the new compounds showed CNS MultiParameter Optimization score predictive of desirable ADMET properties and cross the blood–brain barrier, they fail to achieve in vitro metabolic stability suitable for studies in vivo. The only exception is compound **9b** that therefore deserves further characterization.

## Figures and Tables

**Table 1:** Lipophilicity (cLogP), CNS MPO, and Metabolic Stability (MS) of the Target Compounds.

Compd		Structure	cLogP ^1^	MPO ^2^	MS ^3^
**8a**	n = 3	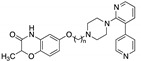	2.79	4.98	4%
**8b**	n = 4		3.31	4.54	14%
**9a**	n = 3	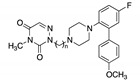	3.06	5.05	<3%
**9b**	n = 4		3.58	4.47	45%
**10a**	n = 3	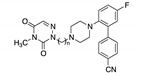	3.08	5.07	5%
**10b**	n = 4		3.60	4.49	26%
**11a**	n = 3	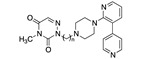	1.24	5.66	4%
**11b**	n = 4		1.76	5.44	3%
**12a**	n = 3	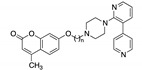	3.55	4.52	19%
**12b**	n = 4		4.07	3.97	14%

^1^ cLogP values were calculated with ChemAxon software; ^2^ Multi-Parameter Optimisation; ^3^ Metabolic Stability (percentage of recovery of the parent compound after incubation for 30 min with rat microsomes).

**Table 2:** Affinity and Cytotoxicity Data of the Target Compounds.

Compd	Affinity							Cytotoxicity
	*K*_i_ [nM] ± S.E.M.			*K*_i_ [nM] (CI 95%)				EC_50_ [μM] ± S.E.M.
	5-HT_1A_^1^	5-HT_2A_^1^	5-HT_7_^1^	D_2_	D_3_	D_1_	D_5_	
**8a**	13.1 ± 2.3	782 ± 119	10.7 ± 0.7	388 (249; 606)	1223 (684; 2185)	6290 (1608; 24,595)	7412 (2038; 26,956)	32.9 ± 3.5
**8b**	17.6 ± 0.9	611 ± 87	9.38 ± 0.42	9.08 (5.07; 16.2)	691 (516; 926)	1711 (981; 2983)	8205 (2475; 27,203)	25.5 ± 2.2
**9a**	653 ± 95	49.1 ± 12	60.8 ± 7.1	>10,000	>10,000	193 (137; 271)	413 (199; 856)	nd
**9b**	23.9 ± 4.9	39.4 ± 7.4	45.0 ± 3.4	>10,000	>10,000	88.3 (35.7; 218)	222 (76.0; 646)	51.9 ± 4.3
**10a**	1091 ± 154	96.8 ± 11	45.7 ± 1.3	>10,000	>10,000	104 (33.0; 331)	180 (96.2; 338)	28.2 ± 3.2
**10b**	43.4 ± 6.2	80.7 ± 3.1	36.5 ± 5.2	>10,000	>10,000	140 (73.4; 268)	101 (33.8; 299)	34.3 ± 2.5
**11a**	269 ± 18	>10,000	44.5 ± 2.2	>10,000	>10,000	>10,000	>10,000	>100
**11b**	5.19 ± 0.12	>10,000	79.4 ± 9.0	>10,000	>10,000	>10,000	>10,000	>100
**12a**	41.5 ± 3.0	315 ± 44	42.5 ± 6.2	300 (210; 430)	1075 (651; 1776)	2198 (780; 6191)	>10,000	35.2 ± 4.1
**12b**	11.3 ± 0.4	~1419 ^2^	52.2 ± 10.6	17.0 (4.56; 63.5)	424 (201; 960)	1527 (354; 6586)	2733 (683; 10,929)	47.9 ± 3.5

^1^ Average data from n = 3 independent experiments performed in duplicate. ^2^ Full displacement of specific binding not achieved at the maximum concentration assayed (100 μM), so *K*_i_ value might not be accurately estimated; maximum displacement achieved was 84%).

## Data Availability

Not applicable.

## Sample Availability

Samples of all target compounds are available from E.L. and M.L.
